# An alert on the incautious use of herbal medicines by sub-Saharan African populations to fight against the COVID-19

**DOI:** 10.11604/pamj.supp.2020.35.2.23161

**Published:** 2020-05-07

**Authors:** Jan René Nkeck, Edmond Elisée Tsafack, Aude Laetitia Ndoadoumgue, Francky Teddy Endomba

**Affiliations:** 1 Faculty of Medicine and Biomedical Sciences, University of Yaoundé I, Cameroon; 2 Ecole des Hautes Etudes en Santé Publique, Rennes, France; 3 Psychiatry Internship Program, University of Bourgogne, 21000 Dijon, France

**Keywords:** Herbal medicines, Covid 19, sub-Saharan Africa

## To the editors of the Pan African Medical Journal

Populations worldwide are currently facing one of the biggest pandemics in history, the coronavirus disease 2019 (COVID-19) pandemic which started in Wuhan (China). Although having declared its first cases several weeks after, Africa, and especially sub-Saharan Africa is actually witnessing an epidemiological progression of COVID-19 within its populations [1]. In parallel with the pandemic´s evolution, various therapeutics are being studied around the world, but at this time none has been uniformly approved. In this context, for preventive and/or therapeutic purpose against COVID-19, some populations notably in sub-Saharan Africa, frequently and firstly resort to traditional herbal medicines, chiefly those known for their respiratory and/or anti-viral therapeutic properties. This is all the more important to note considering the abundance of medicinal plants in sub-Saharan Africa and the low literacy levels of a great part of the population at risk. However, this incautious use of herbal medicines may have reported and detrimental health consequences.

There is a substantial number of plants containing products with potential effects on flu-like diseases, of which we can cite garlic (Sollium sativum), ginger (Zingiber officinalis), ginseng (Panaxia ginseng), echinacea (Echinacea purpurea), eucalyptus essential oil (Eucalyptus globulus), and aloe vera. Otherwise, in sub-Saharan Africa, plants such as Ndole (Vernonia amygdalina), Quinqueliba (Combretum micrathum), Dichrostachys glomerata (used in the drug called APIVIRINE©) and Neem (Azadirachta indica) have little to no known virtues against respiratory diseases but are often mentioned as effective against the COVID-19. Notwithstanding any therapeutic potential, the use of these plants is not without risks especially given that they usually contain several biologically active molecules. For instance, Ginger contains as many as 60 active molecules. The adverse effects of plants could be due to their natural constituents (secondary metabolites), contaminants, degradation products or compounds formed during the process of preparing and preserving the recipe [2,3]. This toxicity may also be related to plants misuse (overdose, drug interactions) or an error in its identification [4]. The secondary metabolites contained in plants are likely to induce significant physiological changes or could interact with an ongoing treatment especially in case of chronic diseases [2]. For example, the consumption of important quantities of garlic can increase bleeding time, decrease plasma concentrations of drugs metabolized by CYP 3A4 (i.e. Saquinavir/Ritonavir) or increase plasma concentrations of those that are substrates of CYP 2E1 (i.e. Paracetamol, Theophylline and Halothane) [5]. Lethal intoxication has been reported after the accidental ingestion of 4-5ml of eucalyptus oil, traditionally used for the treatment of some respiratory conditions [6].

Although the safety profile of plants such as ginseng is fairly good, intoxications remain possible, particularly following contamination with pesticides, aflatoxins, or degradation products, linked to poor harvesting, preparation and/or conservation practices [7]. Aloe vera, whose leaf latex has been widely used for several centuries, should also be used with caution. Indeed, considering its high content in anthraquinone and other polyphenols, its use over a prolonged period (more than two weeks) can lead to potassium depletion; an iterative consumption may cause photosensitization, diarrhea, colic pseudo melanosis, hypertensive crisis, renal insufficiency, hepatic insufficiency, and carcinogenesis [8]. Furthermore, the recognized anti-inflammatory properties of plants such as ginger, eucalyptus oil and Dichrostachys glomerata calls for cautious use, since the benefit/risk balance of anti-inflammatory drugs taken by COVID-19 patients would depend on the type of anti-inflammatory drugs and the stage of the disease [9]. While it is true that the antiparasitic and antimalarial properties of “Ndole” are established, it should be noted that “Ndole” leaves can trigger hypoglycemia especially in cases of uncontrolled consumption [10].

## Conclusion

The therapeutic challenge regarding the current pandemic tends to increase the misuse of traditional herbal medicines in sub-Saharan Africa. However, without evidence-based knowledge of their efficacy on COVID-19, the careless use of medicinal plants can be more harmful than helpful for sub-Saharan African populations. Public health authorities should look into this incautious use and raise populations´ awareness by sharing the right information on each of these plants. Furthermore, it would be of interest that researchers look into clinical trials aiming to assess their efficacy and safety.

## Competing interests

The authors declare no competing interests.
